# Color Change of Phenol Red by Integrated Smart Phone Camera as a Tool for the Determination of Neurotoxic Compounds

**DOI:** 10.3390/s16091212

**Published:** 2016-09-07

**Authors:** Adam Kostelnik, Alexander Cegan, Miroslav Pohanka

**Affiliations:** 1 Faculty of Chemical Technology, University of Pardubice, Studentska 95, Pardubice CZ-53210, Czech Republic; st30827@student.upce.cz (A.K.); Alexander.Cegan@upce.cz (A.C.); 2 Faculty of Military Health Sciences, University of Defense, Trebesska 1575, Hradec Kralove CZ-50001, Czech Republic

**Keywords:** acetylcholinesterase, phenol red, smart phone, drop assay, colorimetry, inhibitor, biosensor

## Abstract

The use of a cell phone as a detection system is easy, simple and does not require trained personnel, which is in contrast to standard laboratory instruments. This paper deals with immobilization of acetylcholinesterase (AChE) in a gelatin matrix, and phenol red, as an indicator of AChE activity, is used in order to establish a method that is easily compatible with a camera device. AChE splits acetylcholine into choline and acetic acid, which changes the pH of a medium, resulting in a phenol red color change. The coloration changed in presence of an AChE inhibitor. Measurements were performed on 3D-printed, tube-shaped holder, and digital photography, with subsequent analysis of red-green-blue (RGB), served for assay purposes. Calibration of AChE inhibitors, tacrine and galantamine, was performed, with limit of detection equal to 1.1 nM and 1.28 µM, respectively. Interferences were also measured, resulting in a proof-of-method stability. The method was further successfully validated for the standard Ellman’s assay, and verified on murine plasma samples spiked with inhibitors.

## 1. Introduction

In the body, the enzyme acetylcholinesterase (AChE) terminates stimulation in a cholinergic system by hydrolysis of acetylcholine into choline and acetic acid [1]. The activity is typically measured in diagnoses or in analytical chemistry for the determination of inhibitors [2,3,4,5]. The most common AChE activity assay is typically based on acetylthiocholine and 5,5’-dithiobis(2-nitro benzoic acid) as a chromogenic reagent, but it can be assayed electrochemically as well [6,7,8]. The acetic acid produced by the aforementioned reaction can also be employed in an AChE activity assay, and acidification of the medium is detected using an acid-base indicator. Several indicators can be used, for example, brilliant yellow, neutral red, phenol red (PR), and so on [9]. Enzyme immobilization is advantageous for its lower costs and the reuse of enzymes [10]. Different matrixes can be used for enzyme entrapment, such as chitosan, alginate, agarose, agar, or gelatin [11,12]. Gelatin is a natural product made of collagen, which undergoes irreversible gelatinization in temperatures under 40 °C [13]. This material has broad biocompatibility and no reported toxicity [14]. Smart phones are widely-spread in the population, and most of them have integrated cameras with high-resolutions, autofocus, and digital zooms [15]. Several applications of smart phones in analytical chemistry, e.g., immunosensors or blood analysis sensors, have been introduced thus far [16]. Digital photography can be evaluated by disparate models, but the RGB channel intensity model is the most common method [17,18,19]. In this work, a biosensor, based on immobilized AChE with colorimetric determination of activity using a camera and phenol red reagent, is proposed for the assay of neurotoxic compounds and is compared to the standard measuring protocol.

## 2. Experimental Section

### 2.1. Materials and Instruments

AChE from electric eel, as a lyophilized powder (≥1000 units/mg protein), acetylcholine chloride (AChCl), acetylthiocholine chloride (ATChCl), 9-amino-1,2,3,4-tetrahydroacridine hydrochloride hydrate (tacrine), galanthamine hydrobromide, tetraisopropyl pyrophosphoramide (iso-OMPA), 5,5’-dithio-bis(2-nitrobenzoic acid) (DTNB), phosphate buffer saline (PBS) pH 7.4, dimethyl sulfoxide (DMSO), Tween-20 and isopropyl alcohol, were purchased from Sigma-Aldrich (St. Louis, MO, USA). Denatured ethanol and gelatin were obtained from PENTA (Prague, Czech Republic). Phenol red (PR) and sodium chloride salt were supplied by ACROS ORGANICS (Thermo Scientific Inc., Waltham, MA, USA). Filter paper (1PS) was obtained from Whatman (Maidstone, UK). Color change was detected using a Sony Xperia MT27i with a 5 Mpx camera and an LED light, using the Android 2.3.7. operating system (device version number 6.0.B.3.184) (Tokyo, Japan). Murine plasma samples were obtained from 20 female BALB/c mice, which were purchased from Velaz (Unetice, Czech Republic). The mice were kept under standard ambient temperature and humidity 50% ± 10%. Light and dark periods lasted 12 h. The mice were sacrificed at the age of 8 weeks, by cutting of the carotid under carbon dioxide narcosis. Blood was taken into lithium heparin treated tubes (Dialab, Prague, Czech Republic) and centrifuged at 1000× *g* for 5 min. Fresh plasma was kept at −80 °C until use in the assay. The whole experiment was both permitted and supervised by the ethical committee of the Faculty of Military Health Sciences (Hradec Kralove, Czech Republic).

### 2.2. Preparation of Gelatin with Immobilized AChE

Gelatin was prepared according the protocol determined by Lourenço et al. [20]. Four-hundred milligrams of powdered gelatin were dissolved in 1 mL of distilled water (60 °C) by stirring for 20 min; then, 2 mL of PBS 7.4 (60 °C) was added and mixture was stirred for another 30 min. After cooling down, the aforementioned mixture was poured into 300 µL of AChE (activity for acetylthiocholine 5.65 × 10^−9^ mol/s/µL) and 300 µL of water and stirred. Then, 50 µL of the final mixture was spread over Whatman filter paper and dried at laboratory temperature for 2 h. Filter paper was stored in a dark box with a saturated humidity of PBS 7.4 at 4 °C overnight.

### 2.3. Solutions Preparation

AChCl solutions were prepared in concentration ranges from 10 mM to 0.16 mM in disposable tubes, and the final volume was set to 1 mL. Tacrine solutions were prepared in concentration ranges from 62.5 to 3.91 nM. Each solution was prepared in PBS 7.4, and the final concentration in drops was 5 times less for AChCl, and 4-fold less in the case of tacrine. Concentration of PR in water was set to 5 × 10^−4^ M. All solutions for the Ellman’s assay were prepared in PBS pH 7.4. DTNB solution was prepared in a concentration of 1 mM and ATChCl, 10 mM. Concentration ranges of tacrine were from 39.1 to 625 nM. Final concentrations in cuvette were 10-fold less concentration of ATChCl and 40-fold less in the case of tacrine. Iso-OMPA was prepared in PBS 7.4 at a concentration of 1 mM; final concentration in plasma samples was 0.1 mM.

### 2.4. Measuring Process

**Figure 1:**
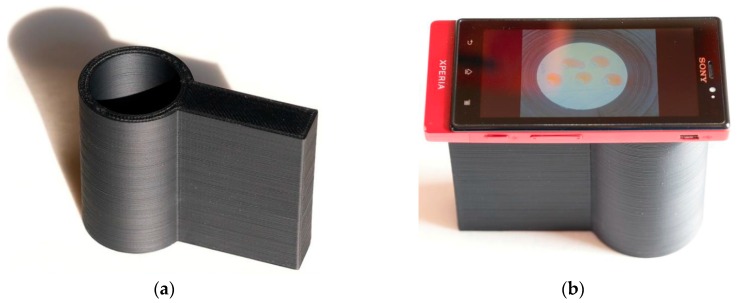
Tube-shaped holder (**a**) prepared using 3D-printing technology, and the final settings for photography (**b**).

Thirty-five microliters of PBS, 25 µL of PBS 7.4 or tacrine solution, 20 µL of PR, and 20 µL of 5 mM AChCl were consequently added to the surface of paper with immobilized AChE. After 5 min of incubation, the surface of the paper was photographed using a smart phone camera placed on a 3D-printed, tube-shaped holder. The holder was printed using a Prusa i3 (Prusa Research, Prague, Czech Republic) using acrylonitrile butadiene styrene shaped into 3-mm filaments. The nozzle temperature was set at 285 °C and the bottom plate temperature was 100 °C during the printing procedure. The individual layers deposited on the final object were 0.1-mm thick. The size of the holder was 80 mm in height, 105 mm in length, and the inner diameter of the tube was 40 mm. Organization of the whole device can be seen in Figure 1.

### 2.5. Ellman’s Assay

Four hundred microliters of DTNB, 25 µL AChE, 25 µL tacrine solution, 450 µL PBS 7.4, and 100 µL of 10 mM ATChCl were added to a standard cuvette. Absorbance was measured at 412 nm immediately and after 5 min of incubation.

### 2.6. Data Processing

Photography was processed in GIMP 2.8.16 (open source software) using the Color Picker function, and RGB colors were obtained. K_M_ value for AChE and ACh as substrates were calculated in Origin software (OriginLab, Northampton, MA, USA) using non-linear curve fitting using the Hill function with the coefficient of cooperativity set to number one. In the Origin software, K_M_ was calculated as the concentration responding to half of the maximal velocity, which was calculated as the upper limit of the curve. Limit of detection for AChE inhibitor tacrine was calculated by linear regression in Origin as a signal to noise ratio equal to three.

## 3. Results and Discussion

**Figure 2:**
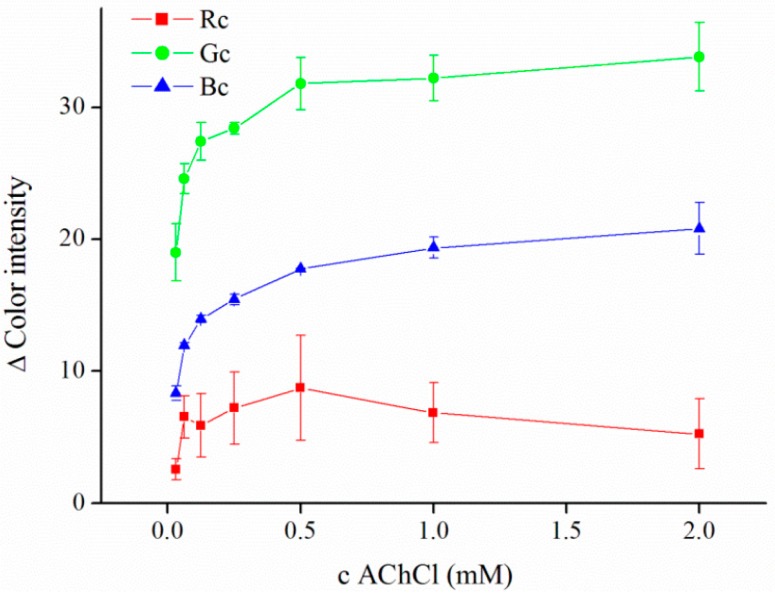
Δ Color intensity for different concentrations of AChCl in all RGB channels. Rc = red channel; Gc = green channel; and Bc = blue channel. Error bars indicate standard error of the mean for *n* = 5.

Mobile phones have proven their ability to serve as detection systems in recent years [16,21,22], and represent a cheaper variant for detection [23]. Colorimetric detection for various molecules [24,25], and also measurement of pH, were introduced [26]. A paper platform offers the possibility of use in the construction of paper-based devices for colorimetric detection [27]. Here, we used paper as a platform for a gelatin matrix and phenol red was used for colorimetric detection. First, we selected the color channel that is suitable for the performance method. Color change in RGB was observed in green and blue channels, the red channel was without change (Figure 2). While the both blue and green channels were suitable for the assay, we decided work with the blue channel further because of the greater difference between maximal and minimal color intensity values. On the other hand, the green channel can be chosen, for example, for reference purposes or the function method whenever necessary.

**Figure 3:**
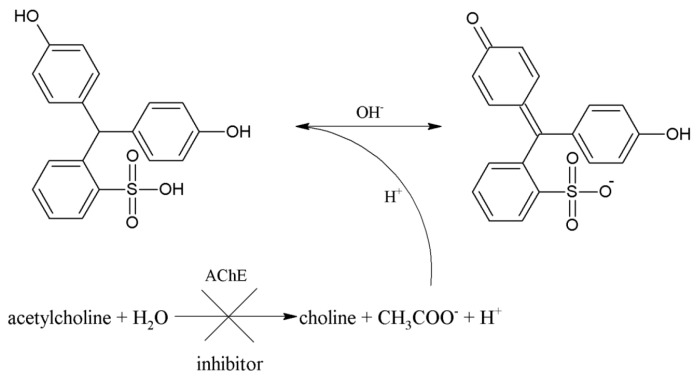
Principle of reaction based on color change of phenol red.

**Figure 4:**
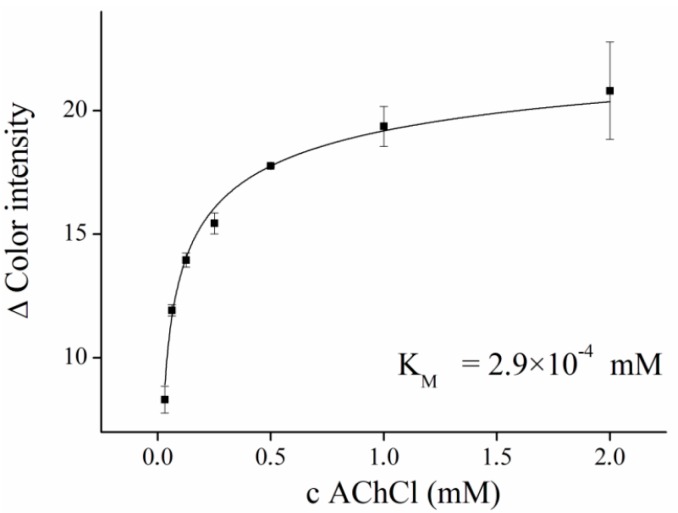
Satuation curve for AChE and acetylcholine as a substrate. The Hill function was used for fitting. Δ Color intensity was observed in the blue channel. Error bars indicate standard error of the mean for *n* = 5.

In neutral pH of PBS 7.4, PR gives a red color. After addition of AChCl, immediately forming acetic acid acidifying medium and the PR turns yellow (Figure 3). We performed a saturation curve for AChE and ACh as a substrate in concentration ranges from 0.031 to 2.00 mM, and the calculated K_M_ value was equal to 2.9 × 10^−4^ mM (Figure 4).

**Figure 5:**
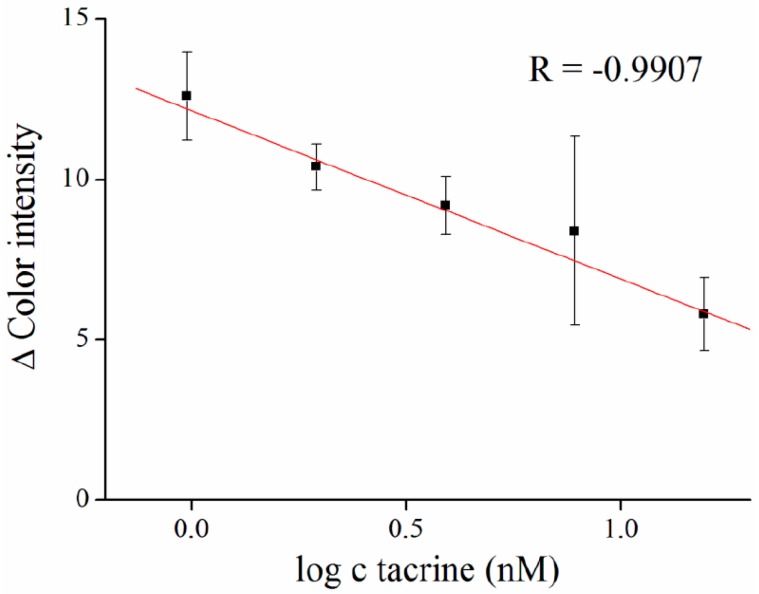
Tacrine calibration curve. Concentration of tacrine is given in logaritmus. Error bars indicate standard error of the mean for *n* = 5.

Gelatin was previously used as a matrix for AChE immobilization in the detection of organophosphates [28] and carbamates [29]. It appears to be a suitable material, which is well penetrable for substrates and inhibitors, but does not affect enzyme activity [30,31,32]. In our work, we used AChE inhibitor tacrine as a model molecule with a high affinity toward the enzyme. We performed a calibration curve in the concentration ranges from 0.98 to 15.63 nM of tacrine, and limit of detection for entrapped AChE was calculated to be equal to 1.1 nM (Figure 5). Comparable results of 10 nM of tacrine, were achieved by Pohanka and Vlcek [33], who measured color intensity using indoxyl acetate.

**Figure 6:**
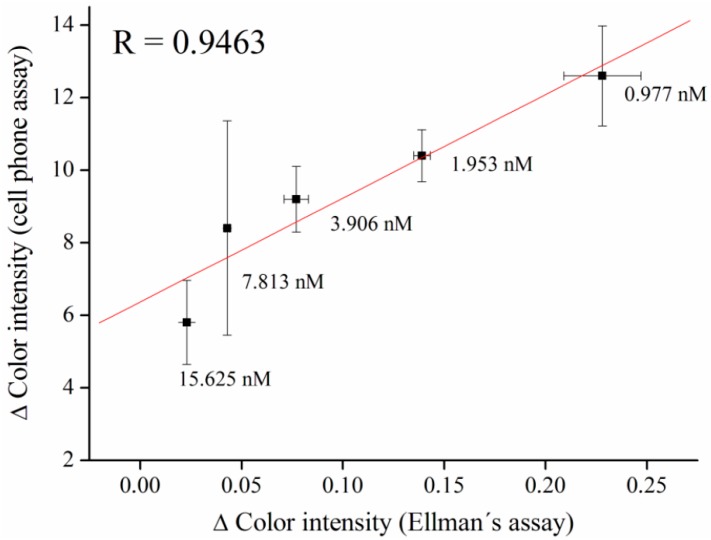
Method validation compared to standard Ellman’s assay. The vertical axis represents Δ Color intensity of the cell phone assay and the horizontal axis represents Δ Color intensity of Ellman’s assay. Error bars for the cell phone assay indicate standard error of the mean and, for Ellman’s assay, a standard deviation for *n* = 5.

The method for tacrine determination was validated, compared to Ellman’s assay and a correlation coefficient of R = 0.9463 was achieved. These results show that analysis using a smart-phone-integrated camera is viable for assay of AChE inhibitors (Figure 6). The experimental data can be extrapolated using a linear model with a good coefficient of determination. Though both methods were equally suitable for the assay, the camera-based assay had several advantages over the standard spectrophotometry test, such as stability of the enzyme and portability of the assay.

**Figure 7:**
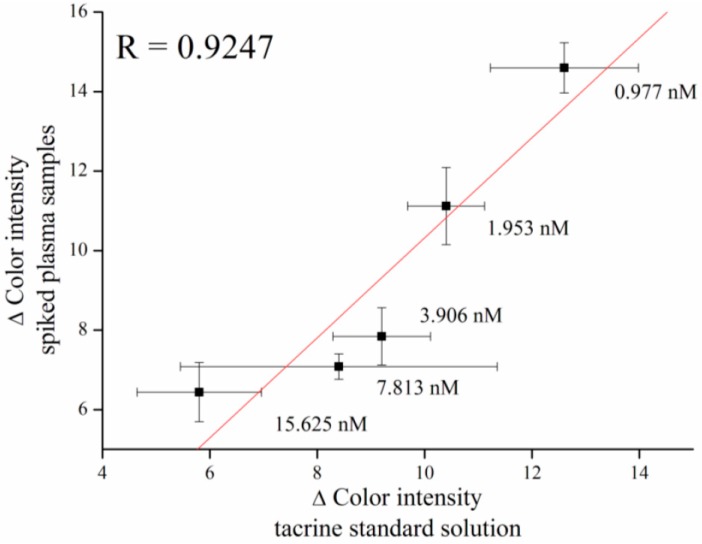
Verifying assay of tacrine in plasma samples compared to a standard tacrine solution. Error bars indicate standard error of the mean for *n* = 5.

For verifying the assay, tacrine was spiked into murine plasma with the same concentrations that were used in the calibration measurement. Butyrylcholinesterase (BChE) naturally occurs in plasma, and because of its affinity towards acetylcholine, inhibition of measurement of AChE activity is required. For this purpose, iso-OMPA, which acts as selective inhibitor of BChE, is used [34]. Results showed feasibility for assay of tacrine with a correlation coefficient of R = 0.9247 (Figure 7).

**Figure 8:**
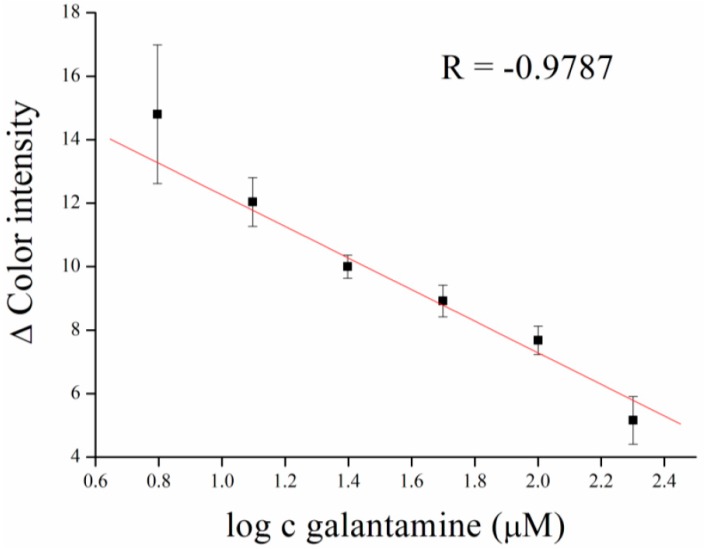
Galantamine calibration curve. Concentration of galantamine is given in logaritmus. Error bars indicate standard error of the mean for *n* = 5.

Calibration for galantamine inhibitor, an anti-Alzheimer drug, was performed as well. Galantamine was measured in concentration ranges from 6.25 to 200 µM, with a limit of detection equal to 1.28 µM (Figure 8).

**Figure 9:**
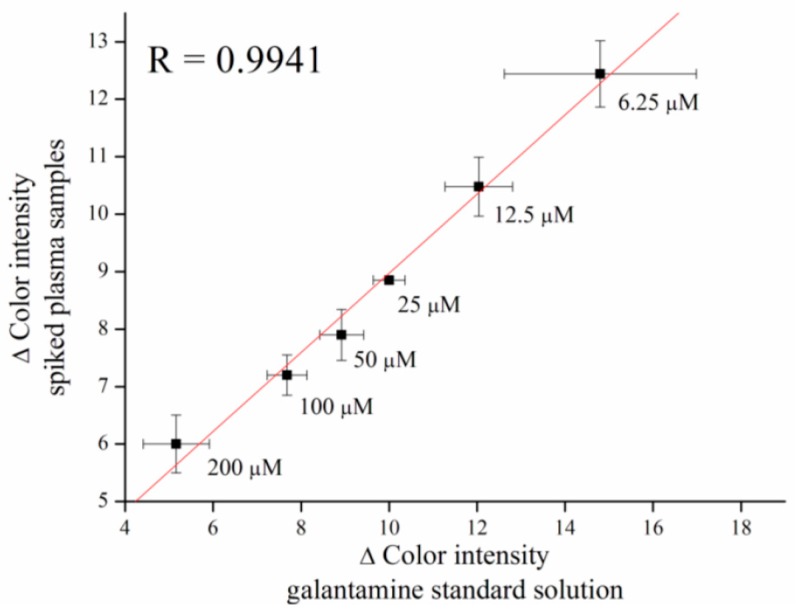
Verifying assay of galantamine in plasma samples compare to standard galantamine solution. Error bars indicate standard error of the mean for *n* = 5.

Verifying of the galantamine assay was performed in murine plasma, as well as tacrine. Galantamine was spiked into plasma samples in appropriate concentrations and a correlation coefficient of R = 0.9941 was achieved (Figure 9).

**Figure 10:**
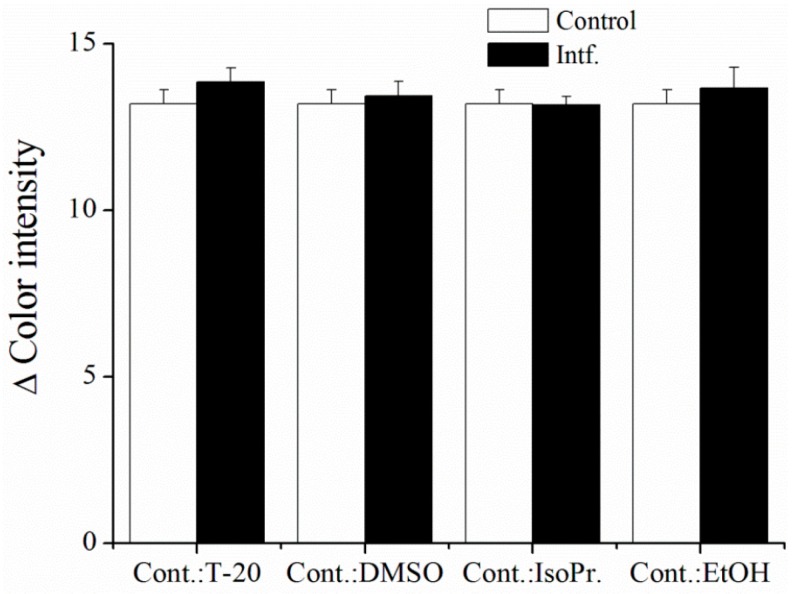
Interferences of organic solvents. T-20 = Tween-20; DMSO = dimethyl sulfoxide; IsoPr. = isopropyl alcohol; EtOH = ethanol; Cont. = control; Intf. = tested organic solvent. Error bars indicate standard error of the mean for *n* = 5.

As was described earlier, the activity of AChE can be reduced using organic solvents, such as methanol, ethanol, and isopropyl alcohol, in small concentration [29]; hence, the organic solvents can be noted as interferents of the AChE-based assay. For the purpose of interference testing, we tested organic solvents DMSO, Tween-20, isopropyl alcohol, and ethanol. All tested solvents were in 5% concentration, except for Tween-20, which was set at 0.25% because of its limited solubility. In the literature, DMSO is known as an inhibitor of AChE, even if present in small concentrations [35], nevertheless it has no effect to the immobilized enzyme. The same conclusion can be made for Tween-20, which also did not inhibit AChE, and this is in compliance with published results [36]. Alcohols are able to inhibit AChE, however, this inhibition occurs in quite high concentrations [29]. We found no interferences toward to AChE activity in the tested concentrations of alcohols. However, isopropyl alcohol caused a decrease of surface tension, which results in a spill of reaction medium (drop) on the paper platform. Ethanol showed the same behavior, but to a lesser extent. This could be used for a tentative resolution of solvent, which was used for the dissolution of AChE inhibitor (Figure 10). We assume that the resistance to organic solvents is caused by the stabilization of the enzyme in the membrane, which protects from denaturation. On the other hand, the phenomenon was not primary aim for our study, and deeper insight should be sought prior to coming to a clear conclusion on this issue.

The presented method is suitable for measurement of AChE inhibitors, and the analysis is quick, has a low cost, and is portable; however, the limit of detection is worse than that of the standard method due to the immobilization of AChE in a gelatin matrix. The fabrication time of the gelatin entrapped enzyme takes around 3 h, but subsequent measurements are quick. A comparison of the presented method with the standard Ellman’s assay and literature are summarized in Table 1.

## 4. Conclusions

Using a smart phone for detection enables a quick and low-cost analysis without the need for sophisticated laboratory methods and trained personnel. We successfully used this method for the determination of AChE activity entrapped in a gelatin matrix. AChE inhibitors tacrine and galantamine were assayed with limits of detection equal to 1.1 nM and 1.28 µM, respectively. Organic solvents were tested for methods interference, and no interference toward AChE activity was observed, while alcohols caused a spill of drops during analysis. The method was correlated with the Ellman’s assay, and results showed that it is usable for the determination of AChE inhibitors. Verifying the assay was performed in murine plasma, and the results showed that the presented method is suitable for measurements in real samples.

## Figures and Tables

**Table 1:** Method comparison with standard Ellman’s assay and literature.

	LOD Achieved	Fabrication Time	Assay Time	Necessary Equipment	Possibility to Check the Assay by a Naked Eye	Determination of Analyte Exact Concentration
Presented camera based assay	Tacrine: 1.1 nM Galantamine: 1.28 µM	3 h	10 min	None—only smartphone	Yes	Yes
Standard Ellman’s assay like here presented	Tacrine: 1.2 pM Galantamine: 18.3 nM	NA	10 min	Spectrophotometer	Yes	Yes
Dipstick assay [37]	Neostigmine, paraoxon: both approx 10^−7^	Aprox. 1 h	45 min	None	Yes	No
Colorimetric assay [33]	Tacrine: 10 nM	Aprox. 1 h	45 min	None	Yes	No
Flow fluorimetric assay [38]	Galantamine: 0.5 µM	NA	10 min	Fluorimeter, pumps, reaction coil	No	Yes
